# 5-Year Survival in Gastric Adenocarcinoma with Epithelial and Stromal Versican Expression

**DOI:** 10.30699/IJP.14.1.26

**Published:** 2018-12-27

**Authors:** Mohammad Hossein Sanei, Omid Mirmosayyeb, Ali Chehrei, Jamshid Ansari, Elahe Saberi

**Affiliations:** 1 *Dept. of Pathology, Isfahan University of Medical Sciences, Isfahan, Iran*; 2 *Students’ Research Committee, Isfahan University of Medical Sciences, Isfahan, Iran*; 3 *Pars Medical Laboratory, Arak University of Medical Sciences, Arak, Iran*; 4 *Dept. of Radiotherapy, Arak University of Medical Sciences, Arak, Iran*

**Keywords:** Stomach Neoplasms Aden carcinoma, Survival, Versican

## Abstract

**Background & Objective:**

Gastric cancer is the second most frequent cause of cancer death worldwide, despite dif- ferences in incidence around the world. The majority of gastric cancer cases concern gastric adenocarcinoma, which has a fairly high 5-year survival rate when coupled with early-stage diagnosis. Versican, a member of the aggregating chondroitin sulfate proteoglycans family, is accumulated predominantly in the tumor stroma. The aim of our study was to investigate versican expression in gastric adenocarcinoma.

**Methods:**

In this study we investigated 80 patients with gastric adenocarcinoma who underwent gastrectomy. Each sample was obtained from paraffin-embedded resected specimens of the stomach after histopathological diagnosis. Patient follow-up was performed every 3 months after the beginning of data collection. Survival analysis was calcu- lated using the Kaplan-Meier method for univariate analysis.

**Results:**

Out of 80 patients with gastric adenocarcinoma, 76 cases (76.3%males and 23.7% females) completed the follow-up period. Positive versican expression in tumor epithelial and stromal cells was found in 39.5% and 22.4% of tumors, respectively. Shorter survival was observed among patients whose gastric adenocarcinoma expressed epithelial or stromal versican.

**Conclusion:**

In summary, the present study suggests that versican is likely a prognostic biomarker that predicts a poor outcome in patients with gastric adenocarcinoma. Comprehensive studies with larger sample sizes are needed.

## Introduction

Gastric cancer is the second most frequent cause of cancer death worldwide, despite extensive differences in incidence around the globe Prevention and personalized treatment are considered as the best options to reduce gastric cancer mortality rates. (1) The majority of gastric cancer cases are of gastric adenocarcinoma, which has a fairly high 5-year survival rate if coupled with early-stage diagnosis. The overall prognosis of gastric cancer is poor, with an average 5-year survival of only 10% to 15%. (2). Clinical prognostic factors such as histological features and tumor staging give limited predictive information for the subsequent treatment of gastric adenocarcinoma. Therefore, new biological markers are in demand to achieve the most effective diagnostic and prognostic method.

The extracellular matrix (ECM), composed of proteoglycans (PGs), glycoproteins and collagens, is a well- organized structure with numerous physiological and pathological roles. (3, 4) Versican, a member of the aggregating chondroitin sulfate PGs family, is accumulated predominantly in the tumor stroma. Due to the specific expressed splice forms of versican, it is able to modulate interactions with the extracellular matrix or neighbor-ing cells. (5)

By increasing the expression of the proteoglycan, versican is strongly associated with poor prognosis for many different cancers. Depending on the cancer type, versican is expressed by either the cancer cells themselves and/ or by stromal cells surrounding the tumor. Versican plays diverse roles in cell adhesion, proliferation, migration and angiogenesis, all features of invasiveness and metastasis. (6) However, the significance of Versican has been less broadly studied in gastric adenocarcinoma, which is why we made it the aim of our study to study its expres- sion in this setting. Furthermore, we determined its relationship with patient survival, placing special emphasis on its prognostic significance.

## Materials and Methods

**Patients**

In this study we investigated 80 patients with gastric adenocarcinoma who underwent a gastrectomy combined with lymph node dissection at Al-Zahra hospital, Isfahan, Iran and Valiasr Hospital, Arak, Iran between 2009 and 2011. This study was approved by the research ethics committee of Isfahan University of Medical Sciences ( approval code: 394623) Inclusion criteria was all patients whose biopsies were confirmed histopathologically as presenting gastric adenocarcinoma according to WHO classifications in the 2010 version of Tumors of Digestive System. Patients who died due to other causes (except gastric adenocarcinoma and its complications) were excluded. Also, patients who did not desire to be followed up until the end of the study were excluded. Informed consent was obtained from each patient before sample collection. Tumor size divided into two categories: less than 6 centimeters and larger than 6 centimeters. Based on pathology reports, tumor differentiation was divided into well, moderate and poor differentiation. The pathological stage was classified according to the eighth American Joint Cancer Committee (AJCC) TNM classification. (7) Baseline characteristics including demographic features and the histological data of all the patients were recorded before enrollment.

**Samples**

Each sample was obtained from paraffin-embedded resected specimens of the stomach after histopathological diagnosis. Then, samples were cut for immunohistochemistry (IHC) staining with anti-versican antibodies (Ab- cam, ab19345). Squamus epithelium and nerve section samples were considered as external and internal positive controls, respectively. Then, they were evaluated based on the immunohistochemical staining. Moreover, ver- sican expression was separately measured in the epithelial and stromal components of the tumor. Morphologi- cal features for the assessment of the epithelial form of versican included the staining of the tumors’ glandular epithelial cells. The stromal form of versican was evaluated based on the staining of surrounding stroma around the glandular tumor cells. The expression was scored according to previous studies, which divided the staining intensity into none (0), mild (1), moderate (2) and intense (3) at low magnification (x100). (8)

**Immunohistochemistry**

Immunohistochemistry for these samples of gastric adenocarcinomas was performed on formalin-fixed, par- affin-embedded, 3-μm-thick tissue sections. Briefly, the sections were deparaffinized three times for 5 min in xylene, and then dehydrated using a graded ethanol series. Endogenous peroxidase activity was halted by the ad- ministration of 3% hydrogen peroxidase and methanol for 5 min. For antigen retrieval, the sections were treated with phosphate-buffered saline (pH :9) at 95°C for 15 min in a microwave oven and allowed to cool for 20 min at room temperature. Subsequently, the sections were incubated at 4°C overnight in a humidified chamber with an anti-versican antibody (Abcam, ab19345) as the primary antibody. The sections were incubated for 45 min at 37°C with a secondary antibody (DAKO real envision). Subsequently, the sections were rinsed three times in PBS and incubated with horseradish peroxidase (DAB + choromogen DAKO) within 5 min for visible light microscopy.

**Follow up**

Patient follow-up was performed every 3 months after the onset of recruitment. According to obtained information, the rates for 1-year, 2 to 3-year and 4 to 5-years survival in patients was determined. Only the patients who completed the 5 years of follow-up were considered in the analysis.

**Statistical Analysis**

All statistical analyses were performed using SPSS (Statistical Package for the Social Sciences, Chicago, IL, USA) version 20. Differences between groups were analyzed using Student’s t-test for continuous variables and the χ2 test for proportions. Survival analysis was calculated using the Kaplan–Meier method for univariate analysis. Multivariate survival analysis was performed by Cox analysis, and in order to control confounding and underlying variables, we used the log-rank test. Variables with a value of (*P*<0.05) were used in univariate analysis, and as such a *P *value of less than 0.05 was considered as being statistically significant.

## Results

**Patient Characteristics**

Out of 80 patients with gastric adenocarcinoma, 76 cases (76% males and 24% females) completed the follow- up period. At the time of diagnosis, 65.8% of cases were less than 70 years old and 34.2% of them were more than 70 years old. The most prevalent tumor stage was stage 3, with a percentage of 51.3%. The percentage of patients with stage 1, 2 and 4 tumors was 15.8%, 31.6% and 1.3% of respectively.

The sites of the tumors were in the body, cardia and antrum in 43.4%, 18.4% and 38.2% of patients. Patients’ baseline characteristics and the tumors’ histopathological details are reported in Table 1.

**Table 1:** Patients characteristics

**Variable**	**n=74**
**Gender**	
Male	57
Female	17
**Age (years)**	
<70	48
≥70	26
**Site of tumor**	
Body	33
Cardia	14
Antrum	27
**Tumor size**	
I	2
II	27
III	45
**Pathological stage**	
I	12
II	23
III	38
IV	1
**Patient status after 5 years**	
Alive	9
Death	65

**Versican expression in gastric adenocarcinoma**

Versican expression was observed in the epithelial and stromal cells of gastric tumors, separately. (Fig- ure-1) Versican was not expressed in 38.1% of tumor samples. Positive versican expression in the epithe- lial and stromal cells of tumors was found in 39.5% and 22.4% of these respective cells. The intensity of staining in positive samples for epithelial versican was mild, moderate and intense in 33.3%, 50.0% and 16.7% of tumors respectively. However, the staining intensity for stromal versican was mild, moderate and intense in 16%, 60% and 24% of tumors respectively.

Versican expression was 43.4%, 18.4% and 38.2% in the body, cardia and antrum of the stomach. Most versican expression in epithelial gastric tumor cells was in the body (50.0%) and most of the expression of versican in stromal gastric tumor cell was in the antrum (41.2%).

**Figure1:**
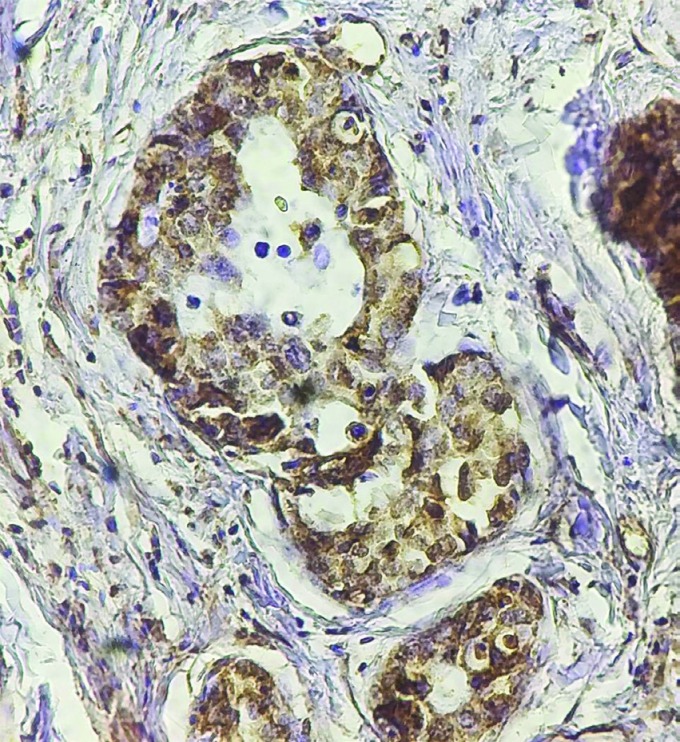
Versican expression as assessed by immunohisto- chemistry in cancerous tissue

**Clinical outcomes**

**Figure 2:**
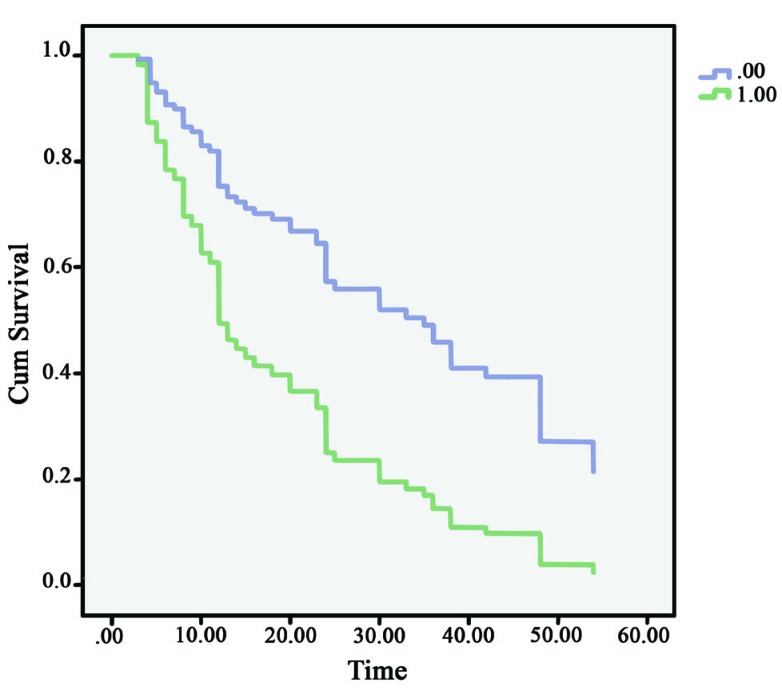
Survival curve of patients according to gastric tu- mor stage. A significant difference was found between tumor stages 1&2 (Mean survival time= 35.5±3.2) and tumor stages 3&4 (Mean survival time= 17.9±2.3). *P*<0.001 [.00: tumor stages 1&2 1.00: tumor stages 3&4]

**Figure 3:**
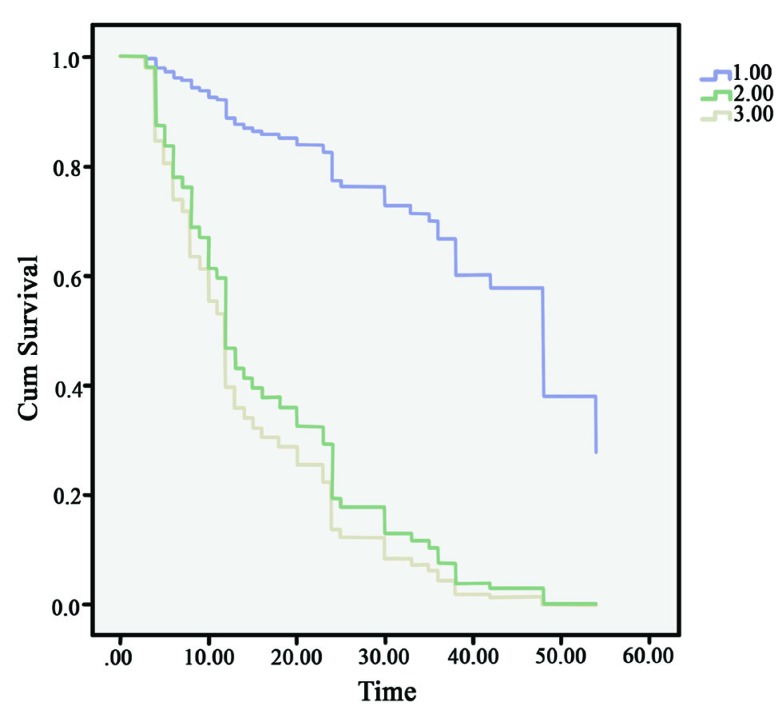
Survival curve of patients according to versican expression of tumor cells. Significantly shorter survival was seen among patients whose gastric adenocar- cinoma expressed epithelial or stromal versican. *P*<0.001 [1.00: no versican expression 2.00: positive for epithelial versican expression 3.00: positive for epithelial versican expression]

The 5-year survival rate of gastric adenocarcinoma was estimated at 12.2% in our study. A lower tumor stage and no expression of versican was correlated sig- nificantly (*P*<0.001) with longer survival. (Figure-2) Significantly shorter survival was seen among patients whose gastric adenocarcinoma expressed epithelial or stromal versican. (Figure-3) Moreover, there was no significant relation (*P*=0.2) between the expression of epithelial or stromal versican and the survival rate.

## Discussion

Invasion and metastasis are characteristics of malignant solid tumors, and many mechanisms are involved in these processes. Cell adhesion molecules, such as integrins, cadherins, and cell-surface heparan sulfate pro- teoglycan, and extracellular matrix components such as versican and syndecan are principally important in the regulation of cell differentiation, morphology and migration. (9) Versican has been detected in many malignan- cies, including melanoma, epithelial ovarian cancer, breast cancer and non-small cell lung cancer. (10-12)

Versican is a large hyaluronan-binding proteoglycan observed in increased quantities in tumor cells. It consists of an N-terminal hyaluronan binding G1 domain, a chondroitin sulfate binding region, and a C-terminal, G3 domain. (6) Elevated levels of versican have been reported in most malignancies, including brain tumors, mela- nomas, osteosarcomas, lymphomas and cancers of the breast, prostate, colon, lung, pancreas, endometrium, and ovary. (13, 14) Versican expression is also associated with cancer relapse and poor patient outcome in breast, prostate and many other cancer types including ovarian cancer. (6) Cultured mammary and prostate fibroblasts produced significant amounts of versican. Furthermore, it was demonstrated that breast and prostate cancer cells could increase versican production by stromal cells. (15)

The present study revealed that the expression of versican in the tumor area predicts a poorer prognosis in gastric adenocarcinoma. Most previous studies have demonstrated that versican expression is predictive of poor prognosis in many cancers, including breast, ovarian, cervical, prostate, endometrial, non-small cell lung cancer and astrocytoma. (16, 17) Thus, the stromal expression of versican has been described as a biomarker for poor prognosis in ovarian cancer, breast cancer and oral squamous cell carcinoma. Epithelial expression has also been reported in endometrial, cervical, ovarian, prostate and colon cancers. On the other hand, the protein expression of versican and lumican predicted good clinical outcomes for stage III and II colon cancer patients, respectively.

(18) Our study showed that the most frequent involved location in patients with expressed epithelial versican was the body. The most frequent location involved in patients with expressed stromal versican was in the antrum. One study in 2001 revealed that an overall difference in survival between the three locations was observed. Cumulative survival was better for patients with carcinomas in the antrum, than in the cardia and in the fundus/ body; no significant differences were observed in the survival between cardia and fundus/body carcinoma cases. Cox regression identified stage and venous invasions as prognostic factors for patients with carcinomas in the three locations. In the group of cardia tumors, older patients had a worse outcome and for fundus/body carcino- mas, large tumors were associated with poorer survival. (19, 20) As the present and other studies have shown, the role of versican in cancer progression is unclear. To our knowledge, the current study is one of the first to clarify the prognostic value of epithelial and stromal versican expression in gastric adenocarcinoma. Our main limitation lay in the low sample size. Significant relations may be seen in studies with larger sample sizes.

## Conclusion

In summary, the present study suggests that versican is likely a prognostic biomarker that predicts a poor out- come in patients with gastric adenocarcinoma. Comprehensive studies with larger sample sizes are essential to investigate the value of epithelial and stromal versican in predicting the prognosis of patients with gastric adenocarcinoma.

## Conflict of Interest

The authors declared no conflict of interests.
